# Multiplex PCR in septic arthritis and periprosthetic joint infections microorganism identification: Results from the application of a new molecular testing diagnostic algorithm

**DOI:** 10.1002/jeo2.12097

**Published:** 2024-07-21

**Authors:** Stefano Ghirardelli, Federica Scaggiante, Christina Troi, Pieralberto Valpiana, Giovanni Cristofolini, Giuseppe Aloisi, Bruno Violante, Arcangelo Russo, Sebastian Schaller, Pier F. Indelli

**Affiliations:** ^1^ Südtiroler Sanitätsbetrieb Brixen Italy; ^2^ Paracelsus Medical University (PMU), Institute of Biomechanics Paracelsus Medical University Salzburg Austria; ^3^ Dipartimento di Medicina Clinica, Sanita' Pubblica, Scienze della Vita e dell'Ambiente Universita' degli Studi dell'Aquila L'Aquila Italy; ^4^ Ospedale Isola Tiberina, Gemelli Isola UOC Chirurgia Protesica e Traumatologica Rome Italy; ^5^ Universita' degli Studi di Enna “Kore” Enna Italy; ^6^ CESAT, Azienda Sanitaria Toscana Centro Fucecchio Italy; ^7^ Department of Orthopaedic Surgery Stanford University School of Medicine Redwood City California USA

**Keywords:** BioFire, DAPRI, multiplex PCR, NGS, periprosthetic joint infections, PJI, septic arthritis, synovial fluid, total knee arthroplasty (TKA)

## Abstract

**Purpose:**

Pathogen identification is key in the treatment of septic arthritis (SA) and periprosthetic joint infections (PJI). This study evaluates the outcome of the application of a new, score‐based SA and PJI diagnostic algorithm, which includes the execution of molecular testing on synovial fluid.

**Methods:**

A score‐based diagnostic algorithm, which includes serologic and synovial fluid markers determination using multiplex PCR (mPCR) and Next Generation Sequencing (NGS) molecular testing, has been applied to a consecutive series of patients with clinically suspected SA or PJI. Patients with a score ≥6 underwent synovial fluid molecular testing, together with traditional culture, to identify the pathogen and its genetically determined antibiotic resistance.

**Results:**

One hundred and seventeen joints in 117 patients (62.5% women; average age 73 years) met the criteria for possible SA/PJI. The affected joint was the knee in 87.5% (joint replacement 66.5%; native joint 21%) and the hip in 12.5% (all replaced joints). 43/117 patients (36.7%) were ultimately diagnosed with SA/PJI. Among the various testing technologies applied, mPCR was the main determinant for pathogen identification in 63%, standard culture in 26%, and mNGS in 11%. *Staphylococcus aureus* and *Enterococcus faecalis* were the top two microorganisms identified by mPCR, while *Staphylococcus epidermidis* was the prevalent organism identified by NGS. mPCR detected the presence/absence of the genetically determined antibiotic resistance of all identified microorganisms. The average timeframe for pathogen identification was 3.13 h for mPCR, 4.5 days for culture, and 3.2 days for NGS.

**Conclusions:**

Molecular diagnostic technologies represent an innovative screening for fast microorganism identification when a joint infection is clinically suspected.

**Level of Evidence:**

Level IV, case series.

**Abbreviations**
AMR: antimicrobial resistance genes
BCB: Blood Culture Bottles
CRP: c‐reactive protein
DAPRI: Debridement, Antibiotic Pearls and Retention of the Implant
ELISA: enzyme‐linked immunosorbent assay
ESR: erythrocyte sedimentation rate
mNGS: metagenomic Next Generation Sequencing
mPCR: multiplex polymerase chain reaction
NGS: Next Generation Sequencing
NIJD: noninfectious inflammatory joint disease
NJI: native joint infection
PJI: periprosthetic joint infection
PMN: polymorphonuclear leucocytes
SA: septic arthritis
TAT: turnaround time
WBC: white blood cells

## INTRODUCTION

Native septic arthritis (SA) and periprosthetic joint infections (PJI) represent, in addition to an economic burden on the healthcare system, a medical emergency due to the risk of septic shock. Pathogen identification is key both in native SA and in PJI. Patients with culture‐negative SA have a longer hospital stay and an increased rate of mortality compared with patients in whom a microorganism can be identified [10]. Regarding PJIs, a recent meta‐analysis did not find differences between culture‐negative and culture‐positive PJI in rates of infection control, periprosthetic, or spacer fracture. However, considering the medical and economic impact of broad‐spectrum antibiotic use, greater efforts should be directed at improving the PJI bacterial identification rate [16, 28]. Unfortunately, culture‐based techniques are challenging, especially when patients have been pretreated with untargeted, broad‐spectrum antibiotics or when difficult‐to‐culture bacteria are encountered [29].

In the post‐SARS‐CoV‐2 era, which has seen the popularization of molecular testing, recommendations from North American [25] and European scientific societies [17] are still based on the use of culture‐based techniques to identify the microorganism causative of a joint infection. This approach has been recently challenged by multiple authors, who have successfully started to utilize molecular testing technologies to identify aseptic arthritis and PJI‐causing microorganisms [5, 26].

Historically, the 2‐stage exchange arthroplasty technique has represented the gold standard in PJI treatment: unfortunately, a 4% perioperative mortality has been reported following its use [14]. As a result, a single‐stage revision approach [3] has become an attractive option: it avoids a second surgery, it is more cost‐effective, and it has shown a success rate similar to the 2‐stage exchange approach [3]. The main requirement for this single‐stage approach is the identification of the causative microorganism, information that has been reported as missing in up to 35% of all PJIs [29]. Recently, an alternative surgical approach to hip and knee PJI is represented by the 1.5‐stage exchange arthroplasty: this surgical technique includes resection of the infected hardware and placement of an articulating spacer placement, usually made by a new femoral component and an all‐poly tibial (in the knee) or acetabular (in the hip) components. It has been recently reported a satisfactory functionality of these kinds of constructs [9], but the two main contra‐indications of this procedure are still represented by the presence of resistant microorganisms and failure to identify the infecting organism [9].

Because of the growing interest in molecular diagnostics in the field of orthopaedic surgery, the current authors recently started to use a molecular testing approach in the setting of an acute PJI, with the goal of promptly identifying the infecting microorganism and saving an acutely infected implant [11]. Following that experience [11], the current authors developed a new molecular testing SA/PJI diagnostic algorithm, which has the primary objective of improving microorganism and antibiotic resistance identification accuracy: this algorithm has been applied to a consecutive series of patients presenting with clinical symptoms of SA/PJI in one of the participating Institutions. The hypothesis of the current study was that a routine inclusion of molecular diagnostics in a step‐by‐step diagnostic algorithm would increase the microorganism identification rate in SA/PJI scenarios: increasing the identification rate could allow for a more targeted antibiotic treatment in the case of SA and could increase the number of single‐stage or 1.5‐stage procedures, with respect to the traditional two‐stage procedures, in a PJI scenario.

## MATERIALS AND METHODS

Only patients reporting symptoms of SA or PJI in the knee or hip and underwent evaluation according to the proposed algorithm were included in the study. Exclusion criteria for performing retrospective analysis were the execution of arthrocentesis in the same joint within 30‐days and a patient age of <18 years. Inclusion criteria were clinical symptoms of SA/PJI: at the time of the medical evaluation, clinical and demographic data were collected, including sex, age range at the time of collection, patients' comorbidities, the location of the infected joint and the presence or absence of an implant.

In total, 117 synovial fluid samples from 117 patients were evaluated according to the diagnostic algorithm: the samples were evaluated at four different institutions that followed the same proposed diagnostic algorithm and protocol.

### Algorithm

**Figure 1:**
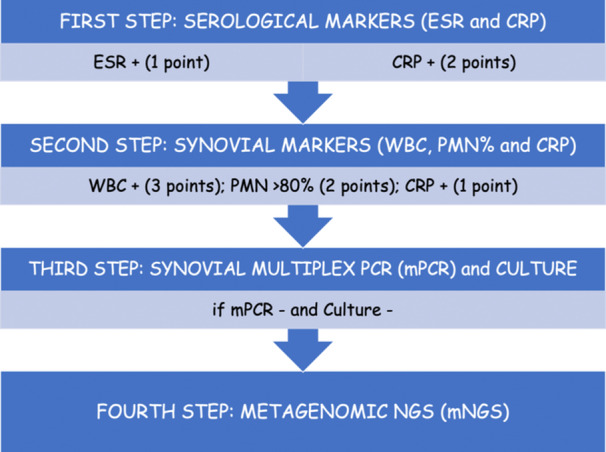
Four‐step diagnostic algorithm for periprosthetic joint infections (PJI). CRP, c‐reactive protein; ESR, erythrocyte sedimentation rate; mNGS, metagenomic next generation sequencing; mPCR, multiplex PCR; PCR, polymerase chain reaction; PMN, polymorphonuclear leucocytes percentage; WBC, white blood cells count.

A new, score‐based diagnostic algorithm (Figure 1) that included serologic and synovial fluid marker determination has been applied to a consecutive series of patients with clinically suspected native hip and knee SA or PJI presenting at four Institutions. The exclusion criteria for this retrospective analysis were previous arthrocentesis in the same joint within 30 days.

The diagnostic algorithm used in this retrospective study has been elaborated according to the SA/PJI diagnostic criteria as presented by the 2018 International Consensus Meeting for Periprosthetic Joint Infections [25] and the European Bone and Joint Infection Society [17]. The algorithm has been completed by the implementation of modern, synovial fluid molecular testing technologies that have been recently recommended by multiple authors [5, 12, 22, 23, 26].

#### Algorithm first step

All patients with a clinical suspicion of knee or hip SA/PJI, who ultimately met the inclusion criteria, underwent serological marker (erythrocyte sedimentation rate or ESR; c‐reactive protein or CRP) evaluation according to Alijanipour et al. [1]. The threshold for ESR at the authors' institution labs was set at 30 mm/h, while the threshold for CRP was set at 1 mg/dL. To assess CRP levels, plasma and synovial fluid were immediately stored in vacuum collection tubes, and synovial CRP was measured using automated enzyme‐linked immunosorbent assay (ELISA) (Thermo Fisher Scientific). Patients with a score ≧ 2, according to the proposed algorithm, were considered at SA/PJI high risk and underwent synovial fluid analysis for white blood cell (WBC) manual count, polymorphonuclear leucocyte percentage (PMN%), and synovial CRP determination (second step).

#### Algorithm second step

The WBC count threshold was set at 3000 cells/μL, the PMN% threshold was set at 80%, and the synovial CRP threshold was set at 6.9 mg/L [1, 20]. Patients with a score ≧ 6 according to the proposed algorithm were considered to have SA/PJI and underwent synovial fluid analysis for microorganism identification, utilizing a commercially available multiplex‐PCR assay (BioFire JI Panel, bioMérieux), and traditional culture (third step).

#### Algorithm third step

The Biofire JI analysis was conducted according to the manufacturer's recommendations. The Biofire JI Panel is characterized by automated nucleic acid extraction, reverse transcription, and nucleic acid amplification. Automated result analysis is approximately elaborated in 1 h per synovial fluid (200 μL minimum) specimen. Organisms and antimicrobial resistance genes (AMR) are qualitatively reported as ‘detected’ or ‘not detected’. This panel can simultaneously identify 31 organisms and up to eight AMR genes. Interestingly, coagulase‐negative staphylococci and Cutibacterium acnes are not represented in the Biofire JI panel. Because of this major limitation, the authors added a fourth step to this protocol, the metagenomic Next Generation Sequencing (mNGS) analysis. The synovial fluid sample was also cultured: it was first inoculated into BD BACTEC Plus Aerobic and Anaerobic Blood Culture Bottles (BCB) (Becton, Dickinson and Company Sparks) and incubated in an automated blood culture system (BACTEC™ FX; BD Diagnostics Systems). The BCBs were processed and tested according to the manufacturer's instructions. Positive blood cultures were analysed using either direct or routine MALDI‐TOF MS. The synovial fluid remained in culture for 14 days.

#### Algorithm fourth step

In mPCR‐negative and culture‐negative joints, the authors performed synovial fluid mNGS analysis (Oxford Nanopore) if the physician in charge of the patient had a high clinical suspicion of SA/PJI due to concomitant factors that can alter the accuracy of the tests performed at the third step (i.e., patient being on antibiotic therapy at the time of culture execution or absence of the infecting micro‐organism in the mPCR panel database). Synovial fluid mNGS has the capability of simultaneously and independently detecting pathogens and multiple target genes in the same clinical, synovial fluid samples without the need for pre‐amplifying target sequences [7].

The final diagnosis as native joint infection (NJI), PJI, or noninfectious inflammatory joint disease (NIJD) was presented according to the algorithm step crucial for microorganism identification and used for final classification. The diagnostic test that ultimately guided the surgical treatment (i.e., arthroscopic irrigation and debridement in case of SA) has been defined as a ‘determinant for identification.’

During the study period, 117 patients (117 joint samples) who entered the second step of the algorithm (after preliminary serological ESR and CRP screening during step 1) met the criteria for possible SA/PJI and were evaluated according to the proposed algorithm: 73 were women (62.5%), and 44 were men with an average age of 73 years (23–87).

#### Statistical analysis

Sensitivity and specificity (including 95% confidence intervals [CI]) were calculated using the MedCalc webpage (https://www.medcalc.org/calc/diagnostic_test.php). All further basic statistical calculations were performed in Excel (Microsoft Office Professional Plus 2013).

## RESULTS

One hundred and seventeen patients (117 synovial fluid samples) entered the study group. Average SA/PJI score according to step 2 of the algorithm was 3.75 (2–9). The affected joint was the knee in 87.5% (presence of a joint replacement in 66.5%; native joint in 21%) and the hip in 12.5% (all total joint arthroplasties).

**Figure 2:**
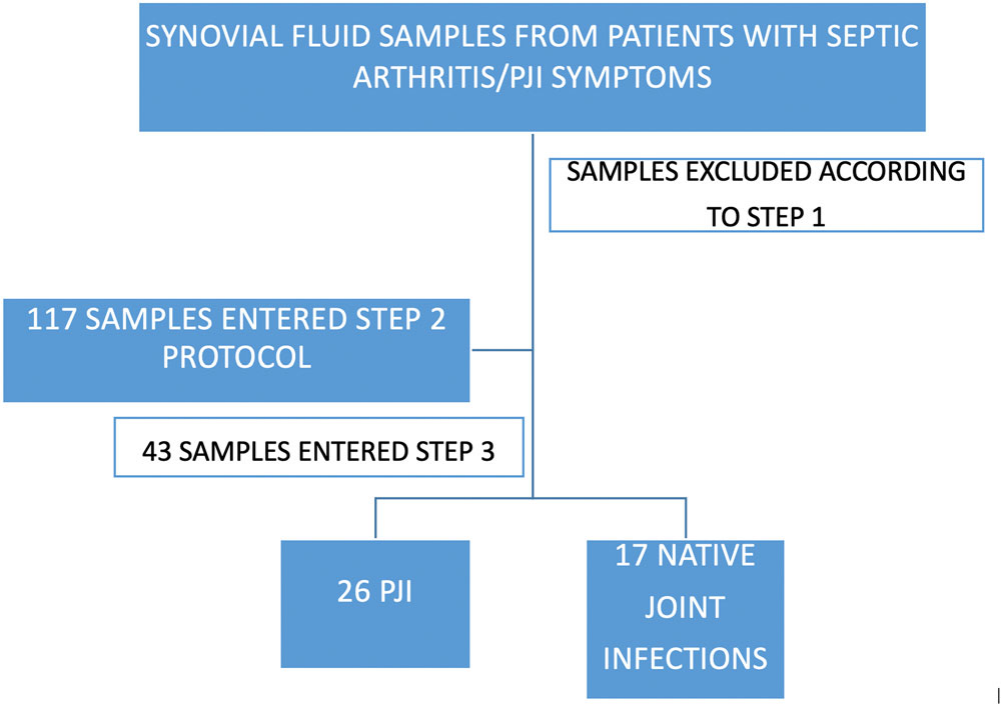
Flowchart of samples collected and included in the study. PJI: periprosthetic joint infections.

Out of the 117 joint samples, 43 (36.7%) underwent molecular testing and culture (step 3) due to a score of ≥6 according to the second step of the proposed algorithm (Figure 2).

**Figure 3:**
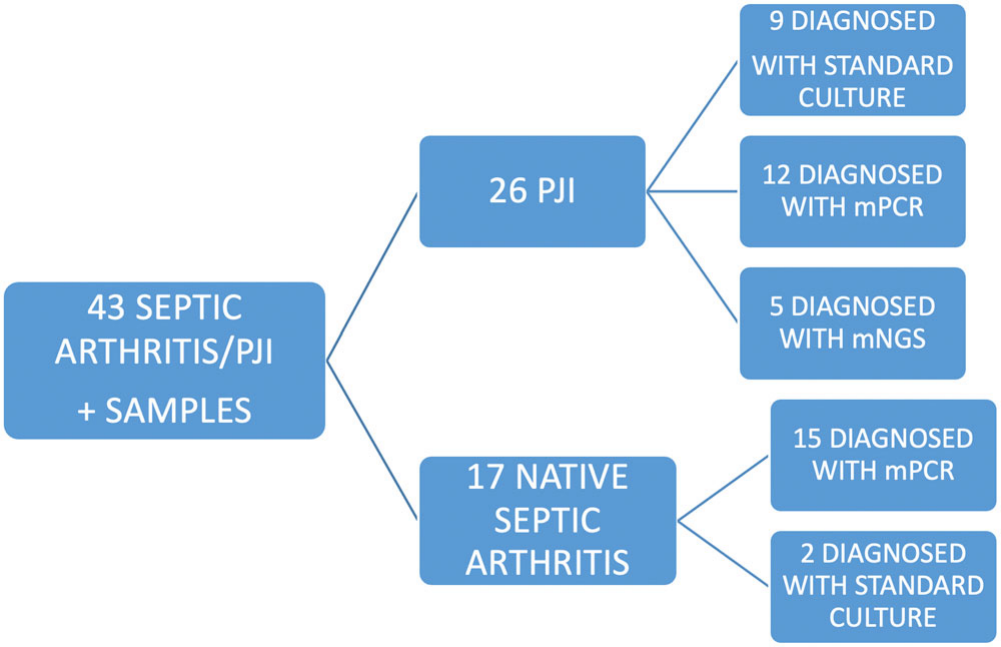
Flowchart of positive samples according to the algorithm. mNGS, metagenomic next generation sequencing; mPCR, multiplex PCR; PCR, polymerase chain reaction; PJI, periprosthetic joint infections.

In this group of 43 samples, the average serologic PCR was 13.50 mg/dL, the average ESR was 51 mm/h, the average synovial WBC count was 83,700, the average synovial PMN was 88%, and the average synovial PCR was 5.7 mg/dL. The microorganism was identified in all 43 synovial fluid samples: mPCR was the main determinant for pathogen identification in 63%, standard culture in 26%, and mNGS in 11% (Figure 3): 19% of all PJI resulted in mPCR‐negative/culture‐negative and were ultimately diagnosed with mNGS.

An overview of the identified microorganisms is reported in Table 1. Seventy‐four samples (73.3%) had a score <6 according to the second step of the algorithm, and those patients were considered to have a noninfectious inflammatory joint disease (NIJD): this diagnosis was confirmed during regular FU examinations.

**Table 1:** Overview of species identified by the application of the algorithm.

Microorganisms N. 43	Joint type	Determinant test for diagnosis
*Staphylococcus aureus* n. 15	Native (8) prosthetic (7/43)	mPCR (13/15) mNGS (2/15)
*Staphylococcus epidermidis* n. 10	Prosthetic (8/43) native (2)	Culture (8/10) mNGS (2/10)
Streptococcus sp. n. 9	Prosthetic (4/43) native (5)	mPCR (9/9)
*Escherichia coli* n. 3	Prosthetic (2/43) native (1)	mPCR (2/3) mNGS (1/3)
*Cutibacterium acnes* n. 2	Prosthetic (2/43)	Culture (2/2)
*Enterococcus faecalis* n. 1	Prosthetic (1/43)	mPCR (1/1)
*S. capitis* n.1	Prosthetic (1/43)	Culture (1/1)
*Enterobacter cloacae* n.1	Native (1)	mPCR (1/1)
*Candida* n.1	Prosthetic (1/43)	mPCR (1/1)

Abbreviations: mNGS, metagenomic next‐generation sequencing; mPCR, multiplex PCR; PCR, polymerase chain reaction.

In the infected group, *Staphylococcus aureus* and *Streptococcus* spp. were the top two microorganisms identified by mPCR, while *S. epidermidis* was the prevalent organism identified by culture and NGS. In the same group, mPCR was able to detect the genetically determined antibiotic resistance of all identified microorganisms. The average timeframe for pathogen identification (turnaround time [TAT]) was 3.13 h for mPCR, 4.5 days for culture, and 3.2 days for NGS. Interestingly, when mPCR was the determinant test for diagnosis, culture TAT averaged 4 days, while when mNGS was the determinant test for diagnosis, culture TAT averaged 5 days.

Four out of 26 PJIs (15.3%) were considered acute (<4 weeks from onset of symptoms), while 18 were considered chronic PJIs. mNGS was the determinant diagnostic test in 11% (5 joints) of the samples (all PJI): the final decision on microorganism identification (2*S. aureus,* 2*S. epidermidis* and 1*E. coli*) was made according to our infectious disease specialists, since mNGS highlighted the presence of five microorganisms for sample.

### Clinical impact

A retrospective chart review of patients in whose synovial fluid microorganisms were detected by either standard culture, mPCR, or mNGS revealed the following surgical treatments:

(1) Irrigation and Debridement of a native joint. The applied algorithm diagnosed 17 cases of SA, with mPCR being the determinant diagnostic test in 88.2% of cases, while the standard culture was only in 11.8%.
(2) PJI. The applied algorithm diagnosed 26 cases of PJI: molecular diagnostics were the determinant diagnostic test in 65.3%. In this PJI subgroup, four cases (15.3%) required a Debridement, Antibiotic Pearls, and Retention of the Implant procedure (DAPRI) [11], 8 cases (30.8%) underwent single‐stage revision, two cases (7.6%) underwent 1.5‐stage revision, while 12 cases (46.3%) underwent two‐stage revision. The DAPRI procedure [11] included several surgical steps: a tumour‐like debridement guided by the methylene blue as the ‘disclosing agent’, an acetic acid irrigation, a 2% chlorhexidine solution intra‐articular scrub, a lavage with a 0.3% diluted povidone iodine wash, irrigation with 9 litres of warm saline solution, a polyethylene insert exchange, and finally, the addition of calcium sulphate antibiotic added beads (Stimulan, Biocomposites).
  Interestingly, 5 out of the 10 cases which ultimately underwent single‐stage or 1.5‐stage revisions were originally culture‐negative: the molecular diagnostic test was able to identify the microorganism, making possible a single‐stage approach instead of a two‐stage approach (standard of care when an infecting micro‐organism is not identified) [17, 20].

## DISCUSSION

To the author's knowledge, this is the first study that reports the preliminary results of a diagnostic algorithm that has included molecular diagnostics as routine testing technology in a PJI‐suspected scenario. The combined use of standard serological markers, standard synovial markers, and modern molecular diagnostic technologies, such as synovial multiplex PCR and synovial NGS, allowed for microorganism identification in all the specimens from joints affected by SA or PJI.

Several recommendations from consensus conferences [20], scientific societies [17], and bone and joint infection registries [21] have already indicated several serological and synovial fluid markers capable of supporting orthopaedic surgeons, microbiologists, and infectious disease specialists in detecting, with high sensitivity and specificity, the presence of native SA or PJI. Unfortunately, there is a lack of similar success in microorganism identification when a joint infection has already been diagnosed. The current literature reports no gold standard test for microbial identification in SA/PJI scenarios, and it has also been reported that 40% of culture‐negative patients ultimately meet the clinical diagnostic criteria for joint infection [13].

The current authors previously implemented molecular diagnostics in the acute phase of a PJI to improve the sensitivity and specificity of routinely recommended serological and synovial markers [11]. The diagnostic algorithm designed and utilized in the current study was based on the application of well‐established serological markers thresholds (ESR and CRP) and synovial fluid biomarkers (WBC count, PMN%, and synovial CRP) to identify patients with an active SA/PJI. The third step of the proposed algorithm included a commercially available syndromic PCR panel able to detect up to 39 potential pathogens and AMR genes directly from synovial fluid. In the current study, the Multiplex PCR was the determinant diagnostic test in 63% of the affected joints: *S. aureus* and Streptococcus species were the most detected microorganisms in this subgroup. Previous large cohort studies showed 100% sensitivity and specificity of the syndromic multiplex PCR [2] to detect in‐panel microorganisms in bacterial arthritis.

Another finding of the current study was that standard culture was the ultimate detection test in only 26% of the cases; this low percentage, compared with mPCR, was due to the intrinsic limitations of conventional cultures: the lengthy time to result, the interference of previous antibiotic treatments, the presence of low virulent pathogens, and ultimately, the low quality of synovial samples [13]. The diagnostic algorithm utilized in the current study included the execution of traditional culture at the same time as mPCR testing: this was done because the current authors still consider culture‐based techniques extremely valuable.

In case of persistent negativity and high suspicion of bacterial arthritis, nucleic acid amplification techniques were utilized in the fourth step of the proposed algorithm. Compared to mPCR, NGS techniques have several theoretical advantages: no limitations on the detection of specific pathogens, allowing thousands or even billions of DNA fragments to be sequenced independently at the same time, and finally, compared with a dedicated pathogen database. NGS techniques demonstrated, in previous studies, very high sensitivity and specificity in microorganism detection [4, 7, 13]. Nevertheless, a major limitation of the use of current NGS techniques in synovial fluid analysis is represented by the high incidence of detection of multiple microorganisms in the same synovial fluid sample: few authors utilized this finding to support the multi‐bacterial etiopathogenesis of many PJI [8, 18] while other authors [27] considered those microorganisms as native microbiome rather than pathogenic microbes. In the current study, mNGS was the main determinant for microorganism detection in only 11%: this finding confirms previous recommendations that nucleic acid amplification techniques do not represent, so far, the first‐line diagnostic technologies for microorganism detection in SA and PJI scenarios [8, 18] but a complementary tool to conventional culture and PCR assays [6, 15].

The major finding on the clinical impact of this study was represented by the fact that, following microorganism identification according to molecular diagnostics, 19.2% of patients underwent single‐stage or 1.5‐stage revision instead of 2‐stage revision. The two‐stage revision has historical downsides, including a prolonged time before final reimplantation, the need for a second hospitalization, and a 25% increased social cost [19] due to a prolonged disability. Because of these limitations, the current and other authors consider one‐stage and 1.5‐stage exchanges as attractive surgical strategies in acute and chronic PJI scenarios.

The current study has several major limitations. First, it is based on the application of a stepwise diagnostic algorithm that has been created according to diagnostic guidelines proposed by multiple authors. The presented algorithm needs to be validated by other studies. The authors acknowledge that, in their algorithm, using similar thresholds for SA and PJI represents a limitation of the study since the current literature has recommended different serological and synovial fluid biomarkers cut‐offs [30]. Second, the decision to continue the diagnostic investigation and move to NGS testing after a negative mPCR result and negative culture during the application of the third step of the algorithm (19% of all PJI met these criteria) has been made by the treating physician based on the presence of clinical symptoms and two assumptions: the culture negativity and the mPCR negativity were determined by a previous assumption of antibiotic (in the case of culture negativity) or by the fact that the infecting microorganism was not present in the mPCR panel. A different physician not having mNGS in his/her diagnostic ‘armamentarium’ could have considered the same patient ‘infection‐free’ instead of ‘false‐negative.’ The fact that the syndromic multiplex PCR test is able to identify only detects and identifies only 31 microorganisms and 8 AMR genes makes this test ideal as a screening test but represents an intrinsic limitation for its wide use. Third, there is a lack of clinical data on all patients. For example, the information on the use of previous antibiotic intakes was not available for all patients, and this could have interfered with the culture‐negativity rate. Fourth, the samples came from four different institutions, and not having a single physician to determine the necessity to move forward in the diagnostic stepwise approach represents a major limitation of the current study. Finally, the patient cohort is small, and a larger number of patients is needed to confirm the study outcomes. The cost of molecular diagnostics in musculoskeletal infection diagnosis is a theoretical limitation to its wider use. Romano et al. [24], in a single‐institution study, hypothesized a € 308 mean direct cost per patient for traditional tissue cultures: this cost has the same order of magnitude as the cost of the mPCR testing utilized in the current study.

Despite these limitations, the strength of the current study lies in the implementation of molecular testing in a standard microbiology workflow, which could be replicated in many general orthopaedic hospitals.

## CONCLUSIONS

To our knowledge, this is the first paper showing the results of a comprehensive stepwise approach that included molecular testing to diagnose native SA or PJI. This algorithm has led to the identification of all the pathogens responsible for the infections, making tailored antibiotic therapy possible for every patient. Further research investigating the clinical results of this approach and its cost‐effectiveness is needed.

## AUTHOR CONTRIBUTIONS

**Stefano Ghirardelli**: Methodology; formal analysis; investigation; writing—original draft preparation. **Federica Scaggiante**: Investigation; resources. **Christina Troi**: Investigation; resources. **Pieralberto Valpiana**: Visualization. **Giovanni Cristofolini**: Validation; visualization. **Giuseppe Aloisi**: Software. **Bruno Violante**: Investigation. **Arcangelo Russo**: Investigation. **Sebastian Schaller**: Project administration. **Pier F. Indelli**: Conceptualization; methodology; formal analysis; investigation; data curation; writing—review and editing; supervision. All authors have read and agreed to the published version of the manuscript.

## CONFLICT OF INTEREST STATEMENT

The authors declare no conflict of interest.

## ETHICS STATEMENT

The study was conducted in accordance with the Declaration of Helsinki and approved by the Institutional Review Board/Ethics Committee of SABES (protocol code 71/2023). Informed consent was obtained from all subjects involved in the study.

## Data Availability

Data that support the findings of this study are available on request from the corresponding author.
